# A Mid-Cycle Rise in Positive and Drop in Negative Moods among Healthy Young Women: A Pilot Study

**DOI:** 10.3390/brainsci13010105

**Published:** 2023-01-05

**Authors:** Ivana Hromatko, Una Mikac

**Affiliations:** Department of Psychology, Faculty of Humanities and Social Sciences, University of Zagreb, 10000 Zagreb, Croatia

**Keywords:** menstrual cycle, positive mood, negative mood, ovulatory shift, prospective study

## Abstract

Clinically oriented studies of mood as a function of the menstrual cycle mainly address the negative moods in the premenstrual phase of the cycle. However, a periovulatory increase in positive emotions and motivations related to reproduction has also been noted. Thus, it has been suggested that the drop in mood during the luteal phase of the menstrual cycle might be a byproduct of elevated positive moods occurring mid-cycle. The aim of this prospective study was to compare both the positive and negative dimensions of mood across the menstrual cycle. A group of 60 healthy, normally cycling women assessed their mood throughout three phases of their menstrual cycles: the early follicular (low estradiol and progesterone), the late follicular (fertile phase; high estradiol, low progesterone) and the mid-luteal phase (high levels of both estradiol and progesterone). Repeated MANOVA evaluations showed a significant increase in positive (friendly, cheerful, focused, active) and a significant decrease in negative (anxious, depressed, fatigued, hostile) dimensions of mood mid-cycle, i.e., during the late follicular phase (η^2^ = 0.072–0.174, *p* < 0.05). Contrary to the widespread belief that negative moods are characteristic of the luteal phase (preceding the onset of the next cycle), the post hoc Bonferroni tests showed that none of the mood dimensions differed between the mid-luteal and early follicular phases of the cycle. The results held when controlling for relationship status and order of testing. This pattern of fluctuations is in accordance with the ovulatory-shift hypothesis, i.e., the notion that the emotions of attraction rise during a short window during which the conception is likely.

## 1. Introduction

There is a widely held belief that most women have elevated negative moods several days before the onset of menstrual bleeding (known as premenstrual syndrome; PMS). However, research in this area does not fully support this notion: a relatively small proportion of women, about 2–6%, can actually be diagnosed with premenstrual dysphoric disorder [1,2,3], which includes changes that are so intense that they disable a person from participating in their usual activities and relationships. These involve regularly occurring physical (e.g., abdominal pain) and psychological (e.g., depression, anxiety, irritability) changes a couple of days before the onset of menstrual bleeding, but not after it [4]. The focus on women who report clinically significant disturbances, and the study designs aiming at assessing only negative emotions and moods, might be driving the findings of many studies into the expected pattern of premenstrual mood dysphoria. However, in a review conducted by Romans et al. [5] it was found that only about 15% of the reviewed studies have shown that a negative mood at the end of the menstrual cycle is more frequent than in other periods of the cycle. At the same time, about 38% of the studies found no evidence of negative mood association with any phase of the menstrual cycle, while another 38% found a negative mood before the menstruation, combined with another phase of the cycle. The finding that 8.5% of the reviewed studies showed a greater negative mood in the non-premenstrual phase only is particularly interesting.

Such a mixed pattern of results can stem from different methodologies, with the retrospective self-reports being especially problematic in this area [6]. When asked to remember how they felt several days before their previous menstrual bleeding, participants were more likely to recall a negative effect, but this pattern disappeared if they were being asked on the exact day, especially if there were not aware of the study aim [7]. Possibly, this is a result of a combination of societal influence, which creates the impression that most women have PMS, as well as imperfections in human cognition, which makes one more inclined to remember and perceive occurrences that are consistent with one’s beliefs (e.g., entering a conflict a few days before the menstrual cycle is interpreted as evidence of PMS) and to forget the ones that do not fit into one’s world view [8]. Some findings suggest that mood depends more upon daily hassles than upon the actual levels of sex hormones [9].

Another potentially confounding methodological aspect is the phase of the cycle to which the premenstrual phase is compared: given the abundance of research showing an ovulatory shift in motives, desires, preferences, and behaviors [10], this issue seems especially important in the light of an evolutionary perspective [11]. The ovulatory-shift hypothesis proposes that women experience evolutionarily adaptive changes in thoughts and behaviors related to mating throughout the menstrual cycle [12,13]. Findings showing that around mid-cycle, women become more active, focused on social events and socializing, feel more attractive, invest more resources in beautifying, flirt more, take more risks, and are sexually more active and more assertive [14,15,16,17,18,19,20,21,22,23,24,25] are in line with the adaptive ovulatory-shift hypothesis. Compared to mid-cycle, in the second half of the cycle, women feel less active and sociable, less attractive, and possibly view changes in their partners’ behavior, involving less attention, jealousy, and protective behavior. Such a change in their own interests and the level of activity might be perceived as a decrease in positive moods. Thus, it could be postulated that during a narrow period of a few days, just before ovulation, positive moods are more frequent, whereas throughout the rest of the menstrual cycle, mood does not oscillate systematically.

Methodological and theoretical confusion notwithstanding, there is ample evidence that variations in levels of estrogen and progesterone do, in fact, influence the affective, cognitive, and motor functioning of healthy women [26,27,28,29,30], as well as their sleep [31] and underlying neural reactivity [32,33,34]. In this study we combined clinical and evolutionary theoretical approaches and analyzed variations in both positive (friendly, cheerful, active, focused) and negative (hostile, anxious, depressed, fatigued) moods as a function of the menstrual cycle among healthy, normally cycling women. To avoid methodological shortcomings of studies calculating proximity to ovulation or proximity to menstruation only (see [35,36]), we chose three time points in the menstrual cycle: the early follicular (low estradiol and progesterone), the late follicular (fertile phase; high estradiol, low progesterone), and the mid-luteal phase (high levels of both estradiol and progesterone). In line with the ovulatory-shift hypothesis, we expected higher scores regarding the positive dimensions of mood in the late follicular phase of the cycle. Based on clinical data, we expected higher scores regarding the negative dimensions of mood in the mid-luteal phase of the cycle. Furthermore, since some studies [22] found that women in relationships tend to be more assertive and independent during the fertile phase, and others [11] suggested that the ovulatory shift pattern would only appear in women who currently exhibited conditions for successful reproduction, we addressed the possible role of relationship status (single vs. in a relationship) in mood fluctuations across the cycle. Finally, we opted to explore the co-variability of shifts in various dimensions of mood. The rationale for addressing this issue was the notion that a deeper understanding of similarities (or a lack thereof) in positive vs. negative mood shifts might provide a useful framework for explaining seemingly discordant findings regarding mood shifts as a function of the menstrual cycle.

## 2. Materials and Methods

### 2.1. Participants and Procedure

G*Power analysis [37] showed that for repeated ANOVA analysis, with three observations, the correlation among the observations was 0.40, the alpha was 0.05, the power was 0.85, and there was a small effect size (f = 0.2); a total sample size of 57 participants was required. Calls for participation were sent via various student online groups and forums. The inclusion criteria were: regular menstrual cycles (not shorter than 24 days or longer than 33 days), no medical condition related to thyroid function or any other endocrine disorder which might influence mood, and no use of oral contraceptives during the six months prior to the study. The health status and regularity of the menstrual cycles of the participants were assessed in an online questionnaire completed at the time of application for participation in the study. A total of 66 healthy (age range: 19–32 years; mean age = 21.8, SD = 2.4), normally cycling women (mean duration of the menstrual cycle = 28.9, SD = 1.9) were recruited. Of them, 53% were single and 47% were in a relationship. The study was conducted online, and all the communication with the participants (instructions, calculations of testing dates, and links to questionnaires) took place via e-mail, upon signing the informed consent. The participants were not rewarded financially, but those of them who were students in the psychology department were given extra course credits. The procedure was in compliance with the Declaration of Helsinki guidelines. The study was approved by the Ethics Committee of the Department of Psychology (Faculty of Humanities and Social Sciences, University of Zagreb; approval code EPOP–2021–020).

Three phases of the menstrual cycle were of interest: the early follicular (low levels of estradiol and progesterone), the late follicular (mid-cycle: high levels of estradiol, low progesterone) and the mid-luteal phase (high levels of estradiol and progesterone). Dates of testing were determined individually for each participant, based on the date of the last menstrual bleeding and the expected date of the next menstruation, using the backward-counting method, which estimates ovulation by subtracting 14 days from the predicted next menses onset. This method is considered to be more reliable than the forward-counting method when using self-reported data [38], and while there are still numerous methodological issues related to the validity of such day-counting methods, in a recent review [39], it was concluded that the backward counting method used in this study (i.e., including the verification of the exact day of the next menstruation and counting backwards to estimate whether the testing was conducted in the correct phase of cycle) exhibited a satisfactory level of validity (0.77, as compared to 0.4–0.55 for forward counting methods).

For each participant, the early-follicular phase session took place between days 3–5 of the cycle (the first two days were avoided, due to the possible effect of menstrual pain or discomfort on mood), the late follicular phase session took place 17–14 days before the expected date of the next menstruation, and the mid-luteal phase session was scheduled 5–8 days before the expected date of the next menstruation. At each of these time points, the participants received an e-mail with a link to the questionnaire and were asked to respond within the next 24 h. Participants were subsequently contacted and asked to report the actual onset of the next menstrual cycle when it occurred, and during this follow up, it was determined that for 6 of them, one or more sessions did not take place in the adequate phase of the cycle (the next menstrual cycle started earlier or later than expected), so their responses were removed from the analyses. The order of sessions was counterbalanced across the participants: for 23 participants the order was FOL, for 20 participants, it was LFO, and for 17 participants, it was OLF (where F = early follicular, O = late follicular/periovulatory, and L = mid-luteal).

### 2.2. Materials

The Adjective Check List (ACL; original instrument: [40]; Croatian translation and validation: [41,42]), a questionnaire comprised of 52 adjectives describing emotional states, was used in this study. Participants had to respond according to how they felt that day, on a scale ranging from 0 (“Not at all”) to 4 (“Extremely”). Average scores were computed for eight dimensions: anxiety (6 items), depression (6 items), friendliness (5 items), joy (5 items), fatigue (8 items), hostility (8 items), concentration (8 items), and energy (6 items). Originally, the list contained 5 additional items pertaining to the response set subscale, but these were omitted from further analyses, as this subscale showed extremely low internal consistency. The alpha coefficients of internal consistency for all other subscales ranged from 0.83 to 0.96.

The rationale for choosing this instrument was twofold: it showed stable psychometric characteristics in numerous studies across various Croatian samples (students, shift workers, etc.), and its scores could be computed separately for each dimension of mood, which was important for testing the specific hypotheses of this study. Dimensions pertaining to social interactions (such as cheerfulness, friendliness, hostility) are particularly informative for exploring the notion of a psychological adaptation making women more likely to socialize and engage in mating behaviors around the time of ovulation.

## 3. Results

### 3.1. Mood across Menstrual Cycle

**Figure 1:**
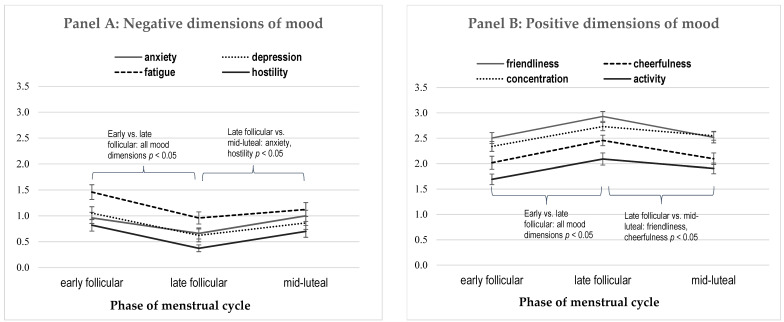
Negative (Panel A: anxiety, depression, fatigue, hostility) and positive (Panel B: friendliness, cheerfulness, concentration, activity) dimensions of mood across the menstrual cycle. Note. Differences between early and late follicular phases were significant for all dimensions of mood; differences between late follicular and mid-luteal phase were significant for anxiety, hostility, friendliness, and cheerfulness; none of the mood dimensions differed between the early follicular and the mid-luteal phase of the cycle.

Repeated measures of MANOVAs, with the phase of cycle as a source of variance within the group, showed significant effects for both positive (*F*(176,8) = 3.19; *p* < 0.002; η^2^ = 0.13) and negative (*F*(176,8) = 2.68; *p* < 0.008; η^2^ = 0.11) moods. Univariate tests for each mood dimension showed a significant effect of cycle (Table 1). As can be seen from Figure 1, showing the average scores and standard mean errors for all mood dimensions across the menstrual cycle, during the late follicular (periovulatory) phase of the cycle, there was a significant increase in the positive dimensions of mood (friendliness, cheerfulness, concentration, energy) and a significant decrease in negative dimensions of mood (anxiety, depression, fatigue, hostility).

Post hoc Bonferroni comparisons showed that none of the dimensions differed between the mid-luteal and early follicular phases of the cycle (see Table 2). Additionally, we repeated the analyses with the order of testing (FOL/LFO/OLF) as a between-participants source of variance: the main effects did not change, and the order of testing was not significant for either positive (*F* = 1.55; *p* = 0.22) or negative (*F* = 2.42; *p* = 0.10) moods. Similarly, the introduction of relationship status (single/in a relationship) as a between-subjects source of variance did not change the reported pattern of results and had no significant main effect for either positive (*F* = 0.16; *p* = 0.69) or negative (*F* = 0.49; *p* = 0.74) moods.

### 3.2. Co-Variability of Various Mood Dimensions

In order to further explore the covariations in cyclical shifts in the various dimensions of mood, we computed a new variable for each mood dimension, defined as a sum of the differences in scores between each of two phases of cycle (y = ∣X_early_follicular_ − X_late_follicular_∣ + ∣X_late_follicular_ − X_mid_luteal_∣ + ∣X_mid_luteal_ − X_late_follicular_∣). As can be seen from Table 3, the covariability of the mood shifts is the largest for shifts in anxiety, depression, and hostility, as well as for activity, cheerfulness, and friendliness. Moderate, but still significant, covariations exist for the majority of other mood dimensions, the only non-significant correlations being among anxiety and fatigue, depression and friendliness, and activity and anxiety. This implies that women differ in amplitudes of mood shifts, and that for most dimensions of mood, those shifts show consistent interindividual variability.

## 4. Discussion

The main finding of this study is that both positive and negative mood dimensions fluctuated throughout the cycle; however, the majority of these changes took place mid-cycle and not at the end of it. This is especially interesting regarding the previous clinical findings of the increased frequency of negative affect in the luteal phase: had we compared the luteal phase with the late follicular phase only, we would have found higher levels of negative affect in the luteal phase, but this would not have revealed the whole picture. Since the post hoc tests showed no differences between the mid-luteal and the early follicular phases, this implies that the negative symptoms lessened in the late follicular phase, and not heightened in luteal phase. In fact, there were no differences in either positive or negative mood dimensions between the early follicular and mid-luteal phases of the cycle. It has been noted previously that the incidence of negative moods in the days preceding the menstrual phase in healthy general population is probably lower than the popular media reports [5].

The specific pattern of fluctuations observed in this study, i.e., the mid-cycle rise in feelings of friendliness, cheerfulness, concentration, and activity, accompanied with a decrease in anxiety, depression, fatigue, and hostility, is in line with the notion of an ovulatory shift, i.e., an adaptive physiological mechanism resulting in heightened overall mood (and thus increased likelihood of interacting with other people) during the narrow window of opportunity when conception is likely. Although we did not measure assertiveness, this pattern is also in accordance with the fertility-assertiveness hypothesis [20,43], postulating that women affect their environment and assert their desires more during the fertile compared to non-fertile phase of their menstrual cycles. Previous studies showed that, during the fertile phase of the menstrual cycle, women engage in more proactive and dominant behaviors, including socializing more and feeling more attractive (e.g., [10,15,20,21,22,23,24,44]. An increase in cheerfulness, friendliness, and activity, accompanied with a decrease in hostility, anxiety, and depression (as observed in this study) would certainly make social interactions easier. Similarly, Romans et al. [45] found that proneness to crying was least likely during the mid-cycle phase, again consistent with the pattern of elevated positive and decreased negative mood. It should be stated that some aspects of the ovulatory-shift hypothesis (namely the ones specifically related to shifts in mate preferences, such as mate’s symmetry and masculinity) were not confirmed, with two separate meta-analyses reaching different conclusions (cf. [10,13]). However, the body of research regarding changes in sexual activity and desire across the menstrual cycle paints a clearer picture, with both older and more recent studies showing increases in either frequency of sexual activity or sexual desire at times of high fertility [14,15,16,17,18,19].

The notion of an evolutionary adaptive hormone-mediated mechanism governing these shifts, and thus increasing reproductive chances, has received a lot of support so far (for a critique of this approach, see [46]), with some researchers arguing that some of the cyclical shifts should be considered spandrels, and not adaptations per se; and others [36] arguing that mood could be a confounder for many of the observed behavioral cyclic changes. The nature of our study does not allow for drawing any conclusions regarding the specific effects of sex steroids, but based on this pattern of results, it could be speculated that in healthy, normally cycling women, without a clinical diagnosis of PMD, variations in estradiol (in addition to variations in progesterone) affect mood fluctuations. More precisely, we speculate that the mid-cycle rise in positive affect could be related to peaking pre-ovulatory estradiol levels [47,48,49,50,51]. Estradiol has been beneficial in the treatment of mood disorders, possibly through its interaction with the serotonin pathways [49,52,53]. Alternatively, one could argue that since pair-bonding behaviors are more intense during ovulation, it is possible that positive affect increases as a consequence of these behaviors [48]. However, a recent study conducted on 384 couples found no compelling evidence that men notice their partners’ fertility status or mid-cycle increases in mate retention tactics [25]. Furthermore, our results showed no effect of being in a romantic relationship on variations in either positive or negative mood. While this does not prove that other sorts of social interactions, which we did not control for, did not have a positive effect on periovulatory mood, the idea that positive mood results in more social interactions seems more plausible.

Additionally, we opted to explore the interindividual variations in the amplitudes of shifts in mood and to test whether there is a systematic covariation between these amplitudes for various dimensions of mood. We found that the covariability of mood shifts is the largest for shifts in anxiety, depression, and hostility, large for activity, cheerfulness, and friendliness, and moderate for the majority of other mood dimensions. The only non-significant correlations we observed were those among shifts in anxiety and fatigue, depression and friendliness, and activity and anxiety. This implies that women differ in the amplitudes of mood shifts, and that for those women who have larger mood shifts across the menstrual cycle, these shifts are consistent across various dimensions of mood. This finding might bear some clinical implications, since the detection of the women who are most susceptible to developing cycle-related mood disorders might include assessment of variations in positive moods as well.

Limitations of the current study. The major limitation of this study is the lack of measurements of hormone levels or ovulation confirmation. Considering the huge interindividual variability of phase lengths and hormonal fluctuations [54], the day counting methods rely on averages which may not reflect the true picture. However, it has recently been shown that even salivary hormone measurements are not as valid indicators as previously thought [55], and at the same time, the backward counting method has been proven to exhibit satisfactory validity [39]. Furthermore, this study did not take into account various other variables which might affect mood, such as daily hassles, social interactions, relationship quality, and dyadic interactions (for those who are in a relationship), etc. Finally, even though this was a prospective study, participants might have been aware of their current cycle phase and could have had certain expectations influencing their self-reports: thus, instead of measuring the emotional status during discrete phases, future studies might avoid this shortcoming by carrying out daily assessments.

## 5. Conclusions

In conclusion, we found a pattern of menstrual cycle-related mood oscilations, which is in accordance with several postulates stemming from the evolutionary psychology theoretical framework. Primarily, we observed a mid-cycle shift toward positive emotions, accompanied with a decrease in negative emotions. Furthermore, it seems plausible that this effect reflects an evolutionary adaptive (hormone-mediated) mechanism, increasing the likelihood of social interactions during the short window of opportunity when conception is most likely. Additionally, we found a systematic co-variability among the amplitudes of mood shifts across the menstrual cycle, meaning that for some women, these shifts shall be larger for a majority of mood dimensions, for both positive and negative moods. Taken together, these findings underline the importance of combining both clinical and evolutionary approaches. The former were traditionaly oriented towards the shifts in negative moods at the end of the cycle, while the latter put forward the notion that some of the variations are adaptive, and some of them might be a by-product or a spandrel. Thus, not all of the menstrual cycle-related oscillations in mood should be considered as symptoms of a mood disorder.

## Figures and Tables

**Table 1:** Results of univariate tests of repeated measures of MANOVA, with phase of menstrual cycle as the within-subjects source of variance, and the dimensions of mood as dependent variables (*N* = 60, *df* = 2).

Mood Dimension	*F*	*p*	Partial Eta Squared
Anxiety	3.436	0.037	0.072
Depression	3.929	0.023	0.082
Fatigue	4.438	0.015	0.092
Hostility	6.986	0.002	0.137
Friendliness	9.290	0.001	0.174
Cheerfulness	6.092	0.003	0.122
Concentration	6.772	0.002	0.133
Energy	3.727	0.028	0.078

**Table 2:** The significance of post hoc Bonferroni comparisons of differences among different phases of the menstrual cycle.

Compared Phases of Cycle	Mood Dimensions							
	Anxiety	Depression	Fatigue	Hostility	Friendliness	Cheerfulness	Concentration	Activity
F-O	**0.035**	**0.006**	**0.004**	**<0.001**	**<0.001**	**0.001**	**0.001**	**0.008**
F-L	0.811	0.252	0.090	0.393	0.856	0.515	0.061	0.159
O-L	**0.013**	0.112	0.285	**0.009**	**<0.001**	**0.010**	0.083	0.102

Note. F = early follicular phase; O = late follicular (periovulatory); L = mid-luteal phase. Significance values less than 0.05 are marked in bold.

**Table 3:** Correlations among total sums of shifts in mood between all three cycle phases.

	Anxiety	Depression	Fatigue	Hostility	Friendliness	Cheerfulness	Concentration	Activity
Anxiety	1	0.690 **	0.275	0.722 **	0.309 *	0.406 **	0.341 *	0.168
Depression		1	0.469 **	0.753 **	0.238	0.471 **	0.582 **	0.373 *
Fatigue			1	0.494 **	0.375 *	0.391 **	0.415 **	0.720 **
Hostility				1	0.428 **	0.512 **	0.511 **	0.383 **
Friendliness					1	0.645 **	0.288 *	0.549 **
Cheerfulness						1	0.394 **	0.650 **
Concentration							1	0.406 **
Activity								1

Note. ** *p* < 0.01; * *p* < 0.05.

## Data Availability

The data presented in this study are available on request from the corresponding author.
